# Chemical composition and biological activities of Salvia officinalis essential oil from Tunisia

**DOI:** 10.17179/excli2016-832

**Published:** 2017-03-06

**Authors:** Med Raâfet Ben Khedher, Saoussen Ben Khedher, Ikbal Chaieb, Slim Tounsi, Mohamed Hammami

**Affiliations:** 1 Laboratory of Biochemistry, 'Nutrition, Functional Food and Vascular Health' Faculty of Medicine of Monastir, University of Monastir, 5000 Monastir, Tunisia; 2 Laboratory of Biopesticides, Centre of Biotechnology of Sfax, University of Sfax, P.O. Box 1177, 3018 Sfax, Tunisia; 3 Unit of Entomology (UR13A-GR09), Regional Research Center on Horticulture and Organic Agriculture (RRCHOA), University of Sousse, Chott-Mariem, 4042, Tunisia

**Keywords:** Salvia officinalis, essential oil, antioxidant, antimicrobial activity, insecticidal activity, phytotoxicity

## Abstract

The aim of this study is to evaluate the chemical composition, antioxidant, antimicrobial, insecticidal and allelopathic activities of Tunisia *Salvia officinalis* essential oil (SoEO). The SoEO was characterized by the presence of 49 components with camphor (25.14 %), α-thujone (18.83 %), 1,8-cineole (14.14 %), viridiflorol (7.98 %), β-thujone (4.46 %) and β-caryophyllene (3.30 %) as the major components, determined by gas chromatography-mass spectrometry. The level of antioxidant activity, determined by complementary tests, namely 2,2-diphenyl-1-picrylhydrazyl radical-scavenging (IC_50_= 6.7 mg/mL), linoleic acid peroxidation (IC_50_= 9.6 mg/mL) and ferric reducing assays (IC_50_= 28.4 mg/mL), was relatively moderate. The SoEO was also screened for its antimicrobial activity. Good to moderate inhibitions were recorded for most of tested microorganisms. It also exhibited important insecticidal activity against *Spodoptera littoralis* larvae and *Tribolium castaneum* adults with LC_50_ values of 55.99 and 97.43 µl/L air, respectively. The effect of the SoEO on seeds germination and growth showed different activities against radical and hypocotyl elongation of the tested species. These results suggest the potential use of the SoEO as natural antimicrobial preservative in cosmetic, pharmaceutical industry and in pest management.

## Introduction

*Salvia* (sage) is the largest genus of the Lamiaceae family, which is native of the Mediterranean area and includes about 900 species (Walker et al., 2004[55]). From its Latin name “*Salvia*”, meaning to cure, it is used in folk medicine for their antibacterial (Özcan et al., 2009[40]), antitumoral (Cardile et al., 2009[7]), antidiabetic (Kim et al., 2007[22]), and antioxidant (Kolak et al., 2009[25]) activities. Sage is also used traditionally in food preparation, herbal tea (Demirci et al., 2005[11]), flavoring agents in perfumery and cosmetics (Delamare Longaray et al., 2007[10]).

In Tunisia, numerous *Salvia* species were investigated. Among them, *Salvia* *officinalis *was encountered in different national parks all along Tunisian territory and considered as a medicinal herb with an interesting essential oil (EO) potential (Chemli, 1997[8]).

Despite the medicinal potential of various plants in Tunisia, at our knowledge, few studies explored the biological activities of this plant. Most of them reported antibacterial, antifungal and antioxidant activities of *S. officinalis* essential oil (SoEO) (Bouaziz et al., 2009[5]; Fellah et al., 2006[15]; Hayouni et al., 2008[20]). Furthermore, there are many reports concerning essential oils from this species in other countries (Delamare Longaray et al., 2007[10]; Tepe et al., 2005[53]). 

These properties make the SoEO very promising as a source of botanical insecticides. This aspect has been largely exploited in the case of EOs from rosemary and other aromatic plants, which are effective against Lepidoptera and Coleoptera larvae (El Abdouni Khiyari et al., 2014[12]; Santana-Méridas et al., 2014[46]). At our knowledge, insecticidal activity of Tunisian SoEO has not yet been investigated. Moreover, allelopathic properties of *Salvia* genus were also reported and the first studies that demonstrated the presence of volatile growth inhibitors produced by *Salvia *species were carried out on *S. leucophylla *and *S. apiana *by Muller and Muller (1964[37]). The effects of EOs extracted from *S. hierosolymitana* and *S. multicaulis *on germination and initial radical elongation of *Raphanus sativus *L. (radish) and *Lepidium sativum *L. (garden cress) showed different activities against the tested species (Mancini et al., 2009[32]), while the phytotoxic activity of the SoEO from Tunisia has not been reported before.

Knowing that the activity of EOs from aromatic plants could be affected by several factors such as the geographical origin, the genetic background of the plant from which the EO was obtained (Pattnaik et al., 1997[41]), the aims of this study were (i) to determine the chemical composition of essential oil extracted from *S. officinalis* from Tunisia (ii) to assess antioxidant activity, (iii) to test antimicrobial activity against some pathogens and phytopathogen strains, (iv) to test insecticidal activity against *Spodoptera littoralis* larvae and *Tribolium castaneum* adults and (v) to evaluate its effects on seed germination and radical and hypocotyl elongation. 

## Materials and Methods

### Plant material

Leaves of *S*. *officinalis* L. were collected in Chott Mariem region located at the Central East of Tunisia, in March 2015. Specimens were identified by Pr. Fethia Harzallah-Skhiri, University of Monastir. A voucher specimen was deposited at the Laboratory of Nutrition - Functional Food & Vascular Health, Faculty of Medicine, University of Monastir, Tunisia and referenced as SO011. The experimental zone is a semi-arid bioclimatic area with a mean rainfall of 450 mm/ year situated at an elevation of 30 m above sea level (latitude 35°54'N, longitude 10°33'E). Particle size analysis of the soil revealed the following composition: silt (14.0 %), clay (12.5 %) and sand (73.5 %). The soil characteristics were: electrical conductivity (2.07 mmho/cm), organic matter (1.65 %), pH (8.38), CaO (1084 ppm), Na (875 ppm), K_2_O (149 ppm) and P_2_O_5_ (48 ppm). 

### Essential oil extraction

SoEO has been extracted from air-dried leaves by hydrodistillation for 3.3 h, using a Clevenger-type apparatus. Five portions (40 g each) of the dried leaves were individually subjected to hydrodistillation. Oil yield was then estimated on the basis of the dry weight of plant material. For antioxidants, antimicrobial, insecticidal and phytotoxic activities, oil was recovered directly, from above the distilled water without adding any solvent; meanwhile for gas chromatography analysis oil was recovered from 1 mL of hexane added above the distilled water. SoEO was stored in opaque glass tubes at -20 °C until use.

### Gas chromatography analysis (GC)

The relative amounts of the SoEO individual constituents were estimated using a Hewlett Packard 5890 II GC (Agilent Technologies, Palo Alto, USA) equipped with Flame Ionization Detector (FID) and HP-5 MS capillary column (5 % phenyl/95 % dimethylpolysiloxane: 30 m × 0.25 mm, *i.d.,* 0.25 μm film thickness). Injector and detector temperature were set at 250 °C and 280 °C, respectively. The oven temperature was kept at 50 °C for 1 min, then gradually raised to 230 °C at 10 °C/min and subsequently, held isothermal for 5 min. Nitrogen was the carrier gas at a constant linear velocity of 38.5 cm/ sec and a flow rate of 1.2 mL/min. The sample was diluted in hexane and 1 µL was injected manually into the system. Quantitative data acquisition of the individual components was electronically obtained from FID area percent data.

### Gas chromatography-mass spectrometry (GC-MS)

The identification of SoEO compounds was performed using a Hewlett Packard 5890 II GC, equipped with a HP 5972 mass selective detector and a HP-5 MS capillary column (30 m × 0.25 mm, *i.d.,* 0.25 μm film thickness). For GC-MS detection, an electron ionization system, with a scan time of 1.5 s, a mass range 40-300 amu (atomic mass unit) and ionization energy of 70 eV, was applied. As a carrier gas, helium was used at a flow rate of 1.2 mL/min. Injector and detector temperatures were set at 250 °C and 280 °C, respectively. The same oven program temperature of GC analysis was used. One micro-liter of diluted samples in hexane was injected manually in the splitless mode. The identification of the compounds was performed by computer matching of mass spectra using library search system HP-5890 (Hewlett-Packard) and consulting data bases of Wiley 275 and NBS 75K libraries (McLafferty, 1989[35]) and NIST 05 (Stein, 1990[51]). Some of the compounds from essential oil were confirmed by comparing their retention times with those of authentic standard substances and with data as reported by Adams (2001[2]).

### Antioxidant activity 

#### DPPH free radical scavenging activity

The ability of SoEO to scavenge the 2,2-diphenyl-1-picrylhydrazyl (DPPH) radical was measured using the method described by Sahin et al. (2004[45]) with modifications. 0.5 mL of different concentrations of SoEO prepared in methanol (1, 5, 10, 15 and 20 mg/mL) was added to 1.5 mL of a methanolic DPPH solution (100 µM). The measurement of absorbance was made against a blank prepared for each concentration at 517 nm after 30 min of incubation in the dark at room temperature. The positive control is represented by a solution of a standard antioxidant (BHT). Its absorbance was measured under the same conditions as the samples. Absorbance values of these solutions were recorded on an ultraviolet and visible (UV-VIS) spectrometer (Lambda 25, PerkinElmer, Inc., Waltham, MA, USA). The results were expressed as percent inhibition (I %) using the following Eq*. *(1):

I % = [(Abs_control_ - Abs_test_) / (Abs_control _- Abs_blank)_] × 100 (1)

where I is the DPPH• inhibition %, Abs_test _is the absorbance value of the essential oil sample, Abs_control_ and Abs_blank_ are the absorbance values of BHT and negative control, respectively. The IC_50_ values were determined graphically by linear regression. All measurements were performed in triplicate and repeated twice.

#### Reducing power determination

The reducing power of SoEO was determined on the basis of the method of Oyaizu (1986[39]). 500 µL of different concentrations of SoEO (1 to 20 mg/mL) were added to 1.25 mL of phosphate buffer (200 µM, pH 6.6) and 1.25 mL of potassium hexacyanoferrate [K_3_Fe (CN)_6_] (10 %). After 20 min at 50 °C, trichloroacetic acid (1.25 mL, 10 %) was added and the mixture was centrifuged at 3000 × *g* for 10 minutes. Finally, 1.25 mL of the supernatant was mixed with 1.25 mL of distilled water and 0.25 mL of ferric chloride [FeCl_3_] (0.1 %). A blank was prepared under the same conditions. BHT was used as the positive control and reading was measured at 700 nm. The experiment was done in triplicate and repeated twice.

#### Linoleic acid peroxidation 

In this assay, the antioxidant capacity of SoEO on inhibition of lipid peroxidation was assessed (Hui et al., 2010[21]). 0.2804 g of Tween 20 and 0.2804 g of linoleic acid were mixed in 50 mL of PBS (200 mM, pH 7.0) to prepare the linoleic acid emulsion. 2.5 mL of linoleic acid emulsion and 2 mL of PBS (200 mM, pH 7.0) were added to 0.5 mL of different concentrations of SoEO (0.1, 0.25, 0.5, 1 mg/mL) and the mixture was incubated for 30 min at 37 °C in the dark. Then, 100 µL of the reaction mixture, 100 µL of ammonium thiocyanate solution (30 %) and 100 µL of ferrous chloride solution (20 mM/ in HCl) were added to 9.7 mL of ethanol (75 %). After stirring for 3 min the absorbance was measured at 500 nm. α-Tocopherol was used as a positive control solution without adding SoEO or α-tocopherol was used as blank. The percentage of antioxidant activity was determined using the following Eq. (2):

Linoleic acid peroxidation inhibition ( %) = [(A_control _-A_test_) / (A_control _-A_blank_)] × 100 (2)

where A_test_ is the absorbance value of SoEO sample, A_control_ is the absorbance of α-tocopherol and A_blank_ is the absorbance of blank.

### Antimicrobial activity 

#### Microorganisms and growth conditions 

Bacteria and fungi strains were obtained from international culture collections (ATCC) and the local culture collection of the Centre of Biotechnology of Sfax, Tunisia. The bacterial strains used in this study included: *Bacillus subtilis*, *Bacillus cereus* ATCC 14579, *Staphylococcus aureus* ATCC 25923, *Micrococcus luteus* ATCC 1880 and Gram-negative bacteria: *Salmonella enterica *serotype *Enteritidis* (*Salmonella enteritidis*; food isolate), *E. coli* ATCC 25922, *Agrobacterium tumefaciens* and the following fungal strains: *Aspergillus niger* CTM 10099, *Aspergillus flavus* (food isolate), *Botrytis cinerea*, *Rhizoctonia solani*, *Fusarium oxysporum* (CTM10402), and *Alternaria alternata* (CTM 10230). 

Bacteria were maintained as stock cultures at -80 °C in Luria Bertoni broth medium (LB), supplemented with 20 % (v/v) glycerol. The fungi were grown on Potato Dextrose Agar (PDA) at 25 °C for 7 days and stored at 4 °C until use. 

The indicator organisms (bacteria) were grown in 3 mL LB broth overnight at 37 °C. For the test, final inoculum concentrations of 10^6^ CFU/mL were used. Fungal spore suspensions were collected from the surface of such fungal colonies by gently scraping with a loop and suspended in 3 mL Potato Dextrose broth (PDB). This suspension was mixed vigorously by vortexing for 15-20 min. The spore suspension stock was diluted to obtain a concentration of 10^6^ spores/mL (measured by Malassez blade). 

#### Agar diffusion method 

The antimicrobial activities of the SoEO were assessed *in vitro* by well diffusion method (Güven et al., 2006[17]), with some modifications. The SoEO was dissolved in ethanol/water (v/v) to a final concentration of 10 mg/mL and then filtered through a sterile Millipore membrane filter (diameter 0.45 µm) and used for activity assay. 100 μL of indicator strain (approximately 10^6 ^CFU/mL) were displayed on plate filled with LB or PDA. Wells (06 mm diameter) were drilled in the agar plates with a sterile Pasteur pipette and then 50 µL of SoEO were added to the wells. The plates were incubated at the optimal temperature of the indicator organism and inhibition zone diameters were measured after appropriate time as described above. Gentamicin (10 μg/wells) was used as a positive control in antibacterial tests, while amphotericin B (20 μg/wells) was used as a positive control in antifungal activity. Negative control consisted of 50 % ethanol which is used to dissolve the SoEO. The experiments were done in triplicate and repeated twice. 

#### Determination of MIC and MMC 

The antimicrobial activity was evaluated by determining the minimum inhibitory concentration (MIC) and the minimum microbicidal concentration (MMC), which includes minimum bactericidal (MBC) and minimum fungicidal concentrations (MFC), using the broth dilution method (Güllüce et al., 2007[16]) with minor modifications against the indicator strains, used in this study. The test was performed in sterile 96-well microplates with a final volume in each microplate well of 100 μL. For susceptibility testing, 90 μL of LB broth or PDB were distributed from the second to the twelfth test wells. A stock solution of the SoEO was prepared by dissolving 100 μL of the oil in ethanol and then adjusted to a final concentration of 50 mg/mL. The first well of the microplate was prepared by dispensing 170 μL of the growth medium and 10 μL of the SoEO to reach a final concentration of 10 mg/mL and then 90 μL of scalar dilutions were transferred from the second to the ninth well. Thereafter, 10 μL of indicator strain (final inoculum concentrations of 10^6^ CFU/mL for bacteria and 10^6 ^spores/mL for fungi) were added to each well. The final extract concentrations adopted to evaluate the antimicrobial activity were 0.039 to 10 mg/ mL. The tenth well was considered as positive growth control containing LB media for bacterial strains while PDB was used for fungi, since no essential oil solution was added. Another well containing 50 % ethanol (v/v), without SoEO, was used as a negative control. The plates were then covered with sterile plate covers and incubated at 37 °C for 24 h for bacterial strains and 72 h for fungi at 28 °C. The MIC was defined as the lowest concentration of the total EO at which the microorganism does not demonstrate visible growth after incubation. As an indicator of microorganism growth, 25 μL of 3-(4,5-Dimethyl-2-thiazolyl)-2,5-diphenyl-2 H-tetrazolium bromide (MTT) (0.5 mg/mL) dissolved in sterile water were added to the wells and incubated at 37 °C for 30 min (Eloff, 1998[13]). When microbial growth was inhibited, the solution in the well remained clear after incubation with MTT. The MMCs were determined by serial subcultivation of 10 μL in LB or PDA plates and incubated at the optimal temperature of the indicator organism. The lowest concentration with no visible growth was defined as the MBC and the MFC, indicating ≥ 99.5 % killing of the original inoculum. 50 % ethanol was used as a negative control. The determinations of MIC, MBC and MFC values were done in triplicate and repeated twice. 

### Insecticidal activity

#### Insect cultures

The insects used in the tests were reared at the Laboratory of Entomology at the Regional Research Center on Horticulture and Organic Agriculture (RRCHOA), University of Sousse, Tunisia. *Spodoptera littoralis* (Lepidoptera: Noctuidae) was reared on artificial diet consisting of a mixture of wheat germ, beer yeast, maize semolina, ascorbic acid, nipagine, benzoic acid, agar and water (Poitout and Bues, 1970[43]) at 26 ± 2 °C, with a photoperiod of 16:8 h light: dark (L:D) and 75 ± 5 % relative humidity (RH) in a growth chamber. The third instar larvae of *S. littoralis *were used for the test. *Tribolium castaneum* (Coleoptera: Tenebrionidae) was reared on wheat flour mixed with yeast (10:1, w/w). The cultures were maintained in a growth chamber at 28 ± 2 °C, with a RH of 75 ± 5 %, in the dark. Only new emerged adults were used for the test. 

#### Bioassays

The insecticidal activity of the SoEO, against the third instar larvae of *S. littoralis* and the adults of *T. castaneum*, was determined by fumigant bioassay using closed container method. A group of 10 larvae or adults were put into the bottom of plastic container of 40 mL. Treatments (concentrations of SoEO 0, 25, 50, 100, 200 and 400 µl/L air) were applied to paper discs, attached at the top of the container, which will be closed. The concentrations quoted above and further in this paper correspond to the volume of the essential oil put on the filter and the volume of the air in the container. Five replicates of each concentration and the control were made. Mortality was recorded after 48 h of the treatment for both *S. littoralis *larvae and *T. castaneum* adults and the fifty percent lethal concentration (LC_50_) was calculated from pooled raw data by probit analysis using programs written in the R. Language (Venables and Smith, 2004[54]).

### Phytotoxicity assay

The biological activity of SoEO was conducted by phytotoxicity assay based on the root/shoot growth and seed germination as described by Moiteiro et al. (2006[36]). Four vegetable species namely, *Triticum aestivum*, *Raphanus sativus*, *Solanum lycopersicum* and *Trigonella foenum-graecum* were selected for this study. Surface-sterilized seeds from each species (*n *= 40) were aseptically transferred on two pre-sterilized layers of Whatman filter paper in petri dishes. After dispensing the essential oil solution to the filter paper, the petri dishes were tightly sealed with parafilm and then incubated at 24 °C, in the dark. Germination was monitored for 7 days and the root/hypocotyl lengths and fresh weights were measured at the end of the experiment (25 digitalized plantlets randomly selected for each experiment) with the application Image J Version 1.37 r, 2010 (http://rsb.info.nih.gov./ij/). 

### Statistical analysis

Data were expressed as means ± standard deviation (SD). The treatments were compared by using analysis of variances (ANOVA). The difference between individual means was deemed to be significant at P < 0.05.

## Results and Discussion

### Chemical composition of S. officinalis essential oil

A total of 49 constituents, representing 97.97 % of the total oil, have been identified from the essential oil extracted from the leaves of *S. officinalis*. In Table 1(Tab. 1) the compounds of the volatile oil and their relative percentages are listed in the order of their Kovats index. The monoterpene fraction of the oil amounted to 75.93 % with oxygen containing monoterpenes as the largest group of this fraction (48.43 %). Camphor (25.14 %), α-thujone (18.83 %), 1,8-cineole (14.14 %) and β-thujone (4.46 %) were the main compounds of the monoterpenoidic fraction. Among the sesquiterpene fraction (17.4 %), sesquiterpene hydrocarbons dominated (9.33 %) with β-caryophyllene (3.30 %) as the major component while viridiflorol (7.98 %) was identified as the main constituent of sesquiterpene alcohol fraction. Other components were present at amount lower than 3 % of the total yield. Our composition differs from that reported by other Tunisian studies, mostly due to the different geographic experimental areas. The EO contents of *S. officinalis* collected from north of Tunisia, were 1,8-cineole (33.27 %), β-thujone (18.40 %), α-thujone (13.45 %), borneol (7.39 %), β-elemene (4.82 %), camphor (3.31 %) and α-pinene (2.74 %) (Hayouni et al., 2008[20]). Fellah et al. (2006[15]) found that SoEO from mountainous region in the center-west of Tunisia was mainly composed by α-thujone (26.49 %), 1,8-cineole (16.96 %), viridiflorol (13,04 %), β-thujone (11.55 %) β-caryophyllene (9.04 %), β-pinene (5.19 %) and camphre (3,38 %). However, essential oil analysis of sage from south of Tunisia revealed that β-thujone (17.76 %), 1,8-cineole (16.29 %), camphor (14.19 %), α-thujone (7.41 %), trans-Caryophyllene (5.45 %) were the major components (Bouaziz et al., 2009[5]). Studies on *S. officinalis* performed in Spain (Laborda et al., 2013[27]), Italy (Marino et al., 2001[34]) and Brazil (Delamare Longaray et al., 2007[10]) also showed a significant variation in the chemical composition of the essential oil of *S. officinalis* compared to our results. 

Compared to the other *Salvia* species, our sage was particularly rich in camphor (25.14 %), α-thujone (18.83 %), 1,8-cineole (14.14 %), viridiflorol (7.98 %), β-Thujone (4.46 %), β-Caryophyllene (3.30 %), borneol (2.81 %), α-Humulene (2.48 %), β-Myrcene (1.93 %), limonene (1.43 %), α-Terpineol (1.33 %) and bornyl acetate (1.05 %). The main characteristic constituents of SoEO found in this study, are in accordance with the profile defined by standard ISO 9909 (Bruneton, 1999[6]) for official *S. officinalis* essential oil, which is cis-thujone (18-43 %), camphor (4.5-24.5 %), cineole (5.5-13 %), humulene (0-12 %), trans-thujone (3-8.5 %), camphene (1.5-7 %), pinene (1-6.5 %), limonene (0.5-3 %), bornyl acetate (2.5 % maximum) and linalool [free and esterified (1 % maximum)].

This variation is probably due to the different growth habitat. Indeed, the oil composition is highly influenced by genetic (Mader et al., 2010[31]), environmental factors (Bettaieb et al., 2009[4]), the developmental stage of the plants and extraction method (Hadri et al., 2010[18]). Previous studies revealed that SoEO composition varies significantly depending on light intensity (Li et al., 1996[29]), soil mineral fertilization (Piccaglia and Marotti, 1993[42]), climate conditions, organ, culture site and season (Santos-Gomes and Fernandes-Ferreira, 2001[47]).

### Antioxidant activity of S. officinalis essential oil

The IC_50_ values (the concentration reducing 50 % of DPPH) obtained for scavenging activity on DPPH radical are shown in Table 2(Tab. 2). According to the recorded results, SoEO exhibited a good antioxidant power with an IC_50 _of 6.7 mg/mL but relatively lower than the synthetic antioxidant BHT used as positive control (IC_50_ = 3.2 µg/mL). The reducing power and the inhibition of linoleic acid peroxidation of SoEO showed a mild to moderate activity compared to the positive controls (Table 2(Tab. 2)). It seems that the antioxidant activity of sage oil is due to the presence of monoterpenes such as α-pinene, a known potent antioxidant (Wang et al., 2008[56]) and several sesquiterpenes (Tamil Selvi et al., 2015[52]) and it is assumed that the contribution of minor and major compounds exhibited this activity and not only one or few active molecules (Wang et al., 2008[56]). 

### Antimicrobial activity of S. officinalis essential oil

The ability of the SoEO to inhibit bacteria and fungi (indicator organisms) was summarized (see Table 3(Tab. 3) and 4(Tab. 4)). Both Gram-positive and Gram-negative bacteria were inhibited by the SoEO. According to MIC and MBC values reported in Table 3(Tab. 3), the SoEO showed an interesting activity against the Gram-positive pathogens (*S. aureus* and *M. luteus*) and also a very good activity against *B. cereus* and *B. subtilis* with MIC values of 0.625 and 0.312 mg/mL, respectively. However, the SoEO cultivated in south Brazil, showed no activity against several *Staphylococcus* strains (Delamare Longaray et al., 2007[10]).

Concerning the Gram-negative bacteria, the sample was slightly active against *E. coli*, *S. enteretidis* and *A. tumefaciens* which showed the lowest values of MIC (2.5 mg/ mL). Our results are similar to those previously reported in the literature, indicating that Gram-positive bacteria are more sensitive to essential oils than Gram-negative bacteria (Mangena and Muyima, 1999[33]). The microorganisms tested in this study cover some human pathogens known as opportunists for man and animals and cause food contamination and deterioration. Our results are of a great importance, particularly in the case of *B. cereus*, *S. aureus* and *S. enteretidis* which are well known for their resistance to a number of phytochemical compounds and for the production of several types of enterotoxins that cause gastroenteritis (Halpin-Dohnalek and Marth, 1989[19]). As shown in Table 4(Tab. 4), the SoEO exhibited varying degrees of antifungal activity against all tested strains. The inhibition zones were in the range of 09 - 15 mm, with MIC values of 0.156 - 5 mg/mL. The most sensitive one was *Fusarium oxysporum* (MIC = 0.156 mg/mL), but the SoEO showed also a very good activity against *Botrytis cinerea* and *Alternaria alternata* (MIC = 0.625 mg/ mL for both). Interestingly, we have demonstrated the capacity of the SoEO to control some fungal strains, particularly fungi (*A. niger*, *A. flavus* and *A. alternata*) responsible for biodeterioration of food during postharvest processing, transport and storage and agricultural phytopathogenic fungi. 

Previous studies reported that there is a relationship between the chemical composition of the most abundant components in the EO and the antimicrobial activity (Deans and Sbodova, 1990[9]). In this context, camphor, α -thujone and 1,8-cineole (herein, abundants in SoEO) are well-known chemicals having antimicrobial potentials (Pattnaik et al., 1997[41]). α-pinene, 2-β-pinene and limonene also had a strong antibacterial activity (Sökmen et al., 2003[50]). These chemical components exerted their toxic effects against microorganisms through the disruption of bacteria or fungal membrane integrity (Knobloch et al., 1989[24]). In addition, the SoEO contained relatively high proportions of oxygenated monoterpenes (Table 1(Tab. 1)) and it is well known that essential oils containing high proportions of oxygenated monoterpenes have strong antifungal activities compared to EO relatively rich in monoterpene hydrocarbons or sesquiterpenes (Kordali et al., 2005[26]). Therefore, the obtained antibacterial and antifungal activities were related to synergistic effects between different major and minor components of the SoEO, suggesting that the SoEO may potentially be useful in food preservation and pest management.

### Insecticidal activity

**Figure 1:**
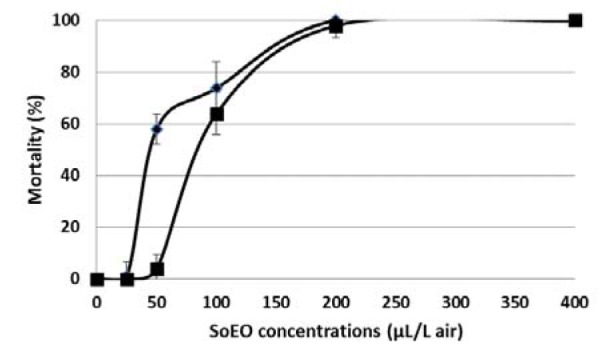
Fumigant activity of *S. officinalis* essential oil against *Spodoptera littoralis* larvae (♦) and *Tribolium castaneum* adults (■) at different concentrations

The SoEO was assayed on *S. littoralis* larvae and *T. castaneum* adults. The percentage of mortality produced by each strain at different SoEO concentrations was shown in Figure 1(Fig. 1). Mortality increased by increasing the SoEO concentration, which revealed a dose-dependent effect. The LC_50_ of SoEO was also calculated for each pest species. The fumigant assay indicated that SoEO showed interesting insecticidal activity against the third instar larvae of *S. littoralis* and adults of *T. castaneum*, with an LC_50_ value of 55.99 ± 7.95 µl/L air and 97.43 ± 11.85 µl/L air, respectively. To our knowledge, there is limited information concerning the activity of SoEO against Lepidopteran larvae *S. littoralis. *Ben El Hadj Ali et al. (2015[3]) reported an approximately similar toxicity exhibited by *Thymus algeriensis* EO against the third instar larvae of* S. littoralis*. The high insecticidal potency of SoEO could be attributed to monoterpenoids compounds such as camphene, α-pinene and γ-terpinene, which were well known for their strong insecticidal activity against *T. castaneum* adults (Kim et al., 2010[23]). In addition, 1-8-cineole and limonene possessed fumigant toxicity against this insect (Lee et al., 2004[28]). These monoterpenoids are known for their inhibitory effects on acetylcholinesterase, a key enzyme in the insect central nervous system (Abdelgaleil et al., 2009[1]; Zarrad et al., 2015[57]). Caryophyllene oxide, a sesquiterpene compound, also showed high fumigant toxicity against this pest. Its high toxicity may result from the inhibition of the mitochondrial electron transport system because changes in the concentration of oxygen or carbon dioxide may affect respiration rate of *T. castaneum*, thus eliciting fumigant toxicity effects (Emekci et al., 2004[14]).

### Phytotoxicity

**Figure 2:**
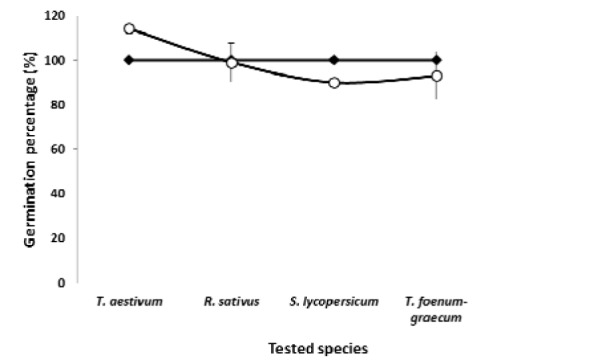
Germination percentages of *T. aestivum*, *R. sativus*, *S. lycopersicum* and *T. foenum-graecum* seedlings. (○) seeds treated with SoEO, (♦) seeds untreated with SoEO. The values in the graphs represent the mean of twenty fives replications ± standard error. Comparison of treated and untreated seeds by SoEO yielded a significant interaction (p < 0.01) between tested species and conditions (with or without SoEO).

**Figure 3:**
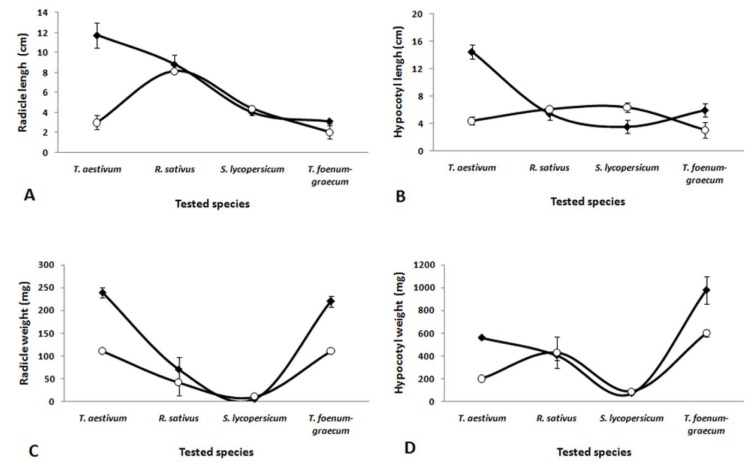
Allelopathic effects of *S. officinalis *essential oil on *T. aestivum*, *R. sativus*, *S. lycopersicum* and *T. foenum-graecum* seedlings. (A) Radicle length, (B) Hypocotyls length, (C) Fresh radicle weight and (D) Fresh hypocotyls weight. The values in the graphs represent the mean of twenty fives replications ± standard error. Comparison of treated and untreated seeds tested by SoEO yielded a significant interaction (p < 0.01) between tested species and conditions (with or without SoEO).

A series of experiments was conducted to assess the level of toxicity of the SoEO on the seed germination, hypocotyl and root lengths and fresh weights of *T. aestivum*, *R. sativus*, *S. lycopersicum* and *T. foenum-graecum* (Figure 2(Fig. 2) and 3(Fig. 3)). The SoEO affected the germination and the seedling growth of tested seeds in a different way. Although the level of the major compounds in the essential oil of *S. officinalis *was closed to those of other* Salvia *species, the interaction between seed species and treatments (with or without SoEO) was statistically significant (P < 0.01). The SoEO did not statistically inhibit the germination of all tested seeds (Figure 2(Fig. 2)). 

Our data disagree with the literature on inhibitory activity exerted by essential oils of *Salvia *species on seed germination (Mancini et al., 2009[32]; Muller and Muller, 1964[37]; Singh et al., 2006[48]). Significant interactions (p < 0.05) between seed species and treatments (with or without SoEo) were also noted in radicle and hypocotyl lengths and weights (Figure 3(Fig. 3)). Indeed, radicle length and hypocotyl length of *T. aestivum* and *T. foenum-graecum* seeds were significantly inhibited by the SoEO, while stimulation was observed on *S. lycopersicum *and *R. sativus *seeds, in comparison to untreated seeds. Fresh weight of roots and hypocotyls of *T. aestivum* and *T. foenum-graecum* also decreased by the SoEO, compared to the untreated seeds (Figure 3(Fig. 3)). 

The inhibition of seedling growth could be attributed to camphor and 1,8-cineole, the main compounds of the SoEO*, *which are potent inhibitors of oxygen uptake by mitochondrial suspensions (Romagni et al., 2000[44]). Moreover, several monoterpenoids of the SoEO, including linalool (Singh et al., 2006[48]), α-pinene (Singh et al., 2006[48]) and limonene (Singh et al., 2006[48]) are potent inhibitors of seedling growth. Previous studies have documented that essential oil and their constituents induce oxidative stress and inhibit root growth (Singh et al., 2006[48], 2009[49]; Mutlu et al., 2011[38]). They reported an enhancement of lipid peroxidation and hydrogen peroxide accumulation and an increase of electrolyte leakage in root tissue (Singh et al., 2009[49]; Mutlu et al., 2011[38]). However, the SoEO does not cause any inhibitory effect on root and hypocotyl elongation and fresh weight on* S. lycopersicum* seeds. López-Iglesias et al. (2014[30]) suggested that enhanced plant growth/plant growth inhibition, could be the result of a positive or negative balance between nutrient and polyphenol concentration. Santana-Méridas et al. (2014[46]) reported a stimulating effect of rosemary residues on *L. sativa *and a moderate phytotoxic effect of *L. perenne*. The phytotoxic effects of the SoEO appeared to be species seeds-dependent.

The results obtained in this study clearly demonstrate that *S.officinalis *essential oil from Eastern Center of Tunisia showed a high variation in its chemical composition compared to those isolated from other country regions. Moderate antioxidant activity of SoEO was demonstrated. This oil exhibited promising antimicrobial and antifungal activities and seems to have a potent fumigant activity against Lepidoptera and Coleoptera pests. Both phytotoxic and stimulating effects on *T. aestivum*, *R. sativus*, *S. lycopersicum* and *T. foenum-graecum* radicle and hypocotyl growth were observed. The high biological activities of *S. officinalis *could be attributed to the components identified by GC-MS analysis. All those results valorize Tunisian *S. officinalis* as a medicinal plant which can be a source of biological active compounds. Therefore, Tunisian *S. officinalis *essential oil could be used as a natural agent in pest management, in cosmetic and pharmaceutical industries.

## Acknowledgement

This study is a part of research program of the Research Laboratory LR12ES05 “Nutrition-Functional food and Vascular Health LR-NAFS” and “DGRST-USCR-Mass Spectrometry” financed by the “Tunisian Ministry of Higher Education and Scientific Research”.

## Conflict of interest

The authors declare that they have no conflict of interest.

## Figures and Tables

**Table 1:**
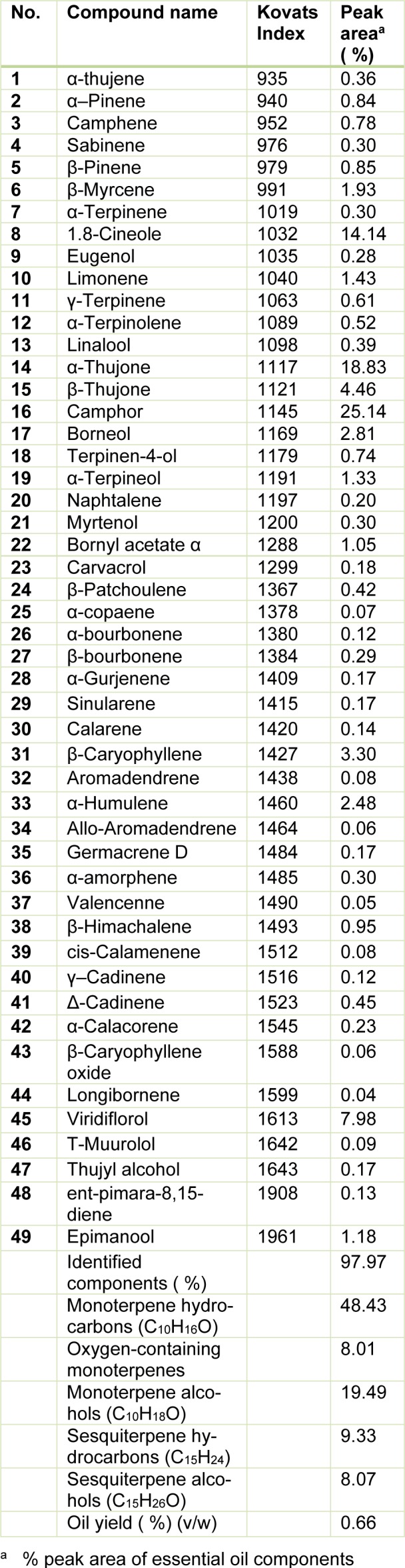
Chemical composition of *S. officinalis* leaves essential oil

**Table 2:**
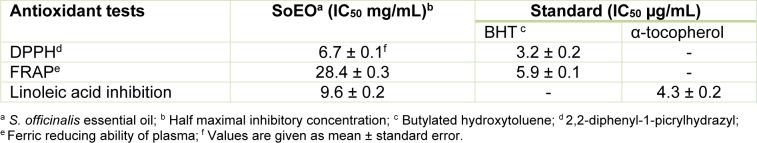
Antioxidant activity of *S. officinalis *essential oil determined by DPPH, FRAP and TBARS test systems

**Table 3:**
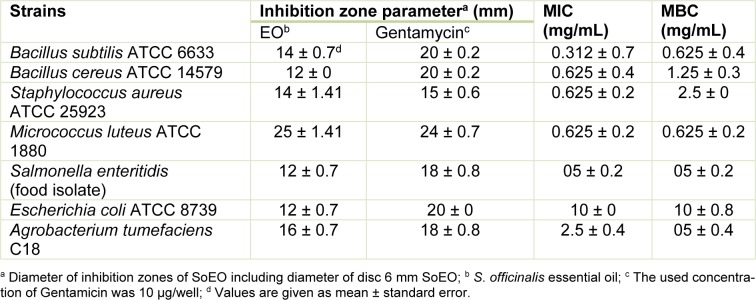
Antibacterial activity of the SoEO against bacteria and determination of the Minimum Inhibitory Concentrations (MICs) and Minimum Bactericidal Concentrations (MBCs) expressed in mg/mL

**Table 4:**
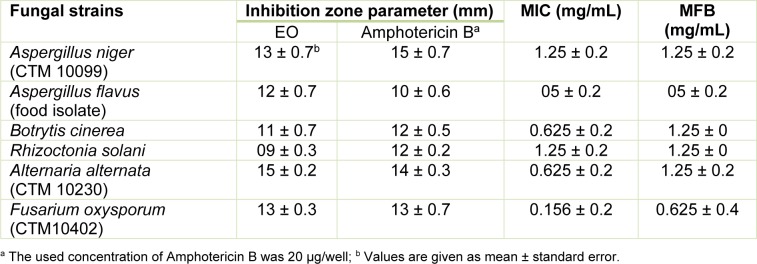
Antifungal activity of the SoEO and determination of the MICs and Minimum Fungicidal Concentrations (MFCs) expressed in mg/mL
